# Anti-counterfeiting system based on luminescent varnish enriched by NIR- excited nanoparticles for paper security

**DOI:** 10.1038/s41598-022-23686-9

**Published:** 2022-11-12

**Authors:** D. Przybylska, T. Grzyb, A. Erdman, K. Olejnik, A. Szczeszak

**Affiliations:** 1 Department of Rare Earths, Faculty of Chemistry, Adam Mickiewicz University in Poznań, Uniwersytetu Poznańskiego 8, 61-614 Poznan, Poland; 2 Centre of Papermaking and Printing, Łódź University of Technology, Wólczańska 221, 93-005 Łódź, Poland

**Keywords:** Chemistry, Engineering, Materials science, Nanoscience and technology, Optics and photonics

## Abstract

Up-converting nanoparticles can be a demand for requirements in many areas, including bioimaging and conversion of energy, but also in the battle against counterfeiting. The properties of lanthanide ions make falsification difficult or even impossible using appropriately designed systems. The proposition of such an approach is the NaErF_4_:Tm^3+^@NaYF_4_ core@shell up-converting nanoparticles combined with transparent varnishes. Given the spectroscopic properties of Er^3+^ ions present in the fluoride matrix, the obtained up-converting nanoparticles absorb light by 808 and 975 nm wavelengths. The intentionally co-doped Tm^3+^ ions enable tuning characteristic green Er^3+^ emission to red luminescence, particularly desirable in anti-counterfeiting applications. The article includes a thorough analysis of structural and morphological properties. Moreover, this work shows that exclusive luminescent properties of NaErF_4_:Tm^3+^@NaYF_4_ NPs can be given to the transparent varnish, providing an excellent anti-counterfeiting system, revealing red emission under two different excitation wavelengths.

## Introduction

Nowadays, exceptional spectroscopic properties of the up-converting nanoparticles (UCNPs) are the answer for biomedicine^1^, optoelectronic^2,3^, and also anti-counterfeiting needs^4–7^. Versatile applications of UCNPs are due to the luminescent properties of the lanthanide ions, Ln^3+^, combined with the up-conversion phenomenon. A non-linear process, an up-conversion emission, involves converting two or more low-energy photons from the near-infrared (NIR) range into one with higher energy. It results in narrow emission bands, relatively long luminescence lifetimes (from µs to ms), low autofluorescence, negligible photobleaching, and a high signal-to-noise ratio^8^. Extra features of the UCNPs, such as high chemical stability and low toxicity, are another advantage, making them extensively applied^9–11^.

The most common and well-known inorganic UC materials are those doped with Ln^3+^, which typically exhibit visible emission activated under a NIR 980 nm excitation wavelength^12^. Such systems contain a pair of Ln^3+^ ions, typically Yb^3+^ ions acting as a sensitizer, and other Ln^3+^ ions (e.g., Er^3+^, Ho^3+^or Tm^3+^ ions), which play the role of emitters^13,14^. This type of luminescent system doped in fluoride materials, AREF_4_ type (where A = Na, Li, K; RE = Y, Lu or Gd), is considered one of the most efficient luminophores^15,16^. However, the brightness and efficiency of the UCNPs mentioned above are limited, determined by low values of dopant ions' concentration, which is 18–20% for Yb^3+^ and generally less than 2% in the case of luminescence activator ions. Higher concentrations result in cross-relaxation processes (CR) and, in general, concentration quenching, thereby decreasing the luminescence efficiency^4,17^. The solution to these limitations is the core@shell structure of the UCNPs, composed of a protective inert shell, which minimizes energy migration to surface defects due to the isolation of activator ions from surface-quenching centers and luminescent cores responsible for the emitting properties^18^. As an example, this strategy allows for reaching the UC quantum yield (UCQY) of around 7.6% for LiLuF_4_/Yb/Tm@LiLuF_4_ (20:0.5) nanoparticles^19^. The same bare nanoparticles, NPs, forming cores, possess a UCQY of only 0.61%^19^. Moreover, the core@shell structure allows a high concentration of sensitizer/emitter ions, significantly improving the observed emission intensity^20,21^.

Although the highly doped core@shell structure solves the problem of migration energy to surface quenchers, the cross-relaxation process can still occur. Fortunately, this phenomenon has bright sides, as it enables the observation of single-band emission, which is favorable in anti-counterfeiting activities for documents or valuable product protection. Chen et al. described the process in detail^22^ and explained the pure red emission for NaErF_4_:Tm^3+^@NaYF_4_ core@shell UCNPs. The structure excludes the presence of Yb^3+^ ions, typically used in the UC system because Er^3+^ ions are simultaneously sensitizers and emitter ions. Er^3+^ ions have the unique property that they can be excited with up to three wavelengths in the NIR range (i.e., by 808 or 980 nm wavelengths) due to the direct absorption. Then, the absorbed energy goes to another co-doped activator, such as Ho^3+^, Nd^3+^, or Tm^3+^ ions^23^. Such sophisticated UCNPs are relevant for anti-counterfeiting of documents and valuable products due to the multimodal security system. It means multi-band excitation and a precise red emission color, which significantly improves the protection of the product. Another advantage of anti-counterfeiting systems based on core@shell fluoride UCNPs ensures obtaining very intense emission due to the relatively low phonon energy of the inorganic matrix. The luminescence intensity is incomparably higher than the emission of other matrices doped with Ln^3+^ ions, for example, those composed of oxygen, e.g., oxides or vanadates matrices^24–27^. In addition, the nanosize and homogeneity of this type of UCNPs prepared by precipitation in the high-boiling solvents method allow easy mixing with many different media, including commercial varnishes. For example, the solid-state reaction process results in bulk materials, and this type of phosphors can not form stable, transparent colloids, only suspension. It means they cannot create a homogenous mixture with inks or varnishes. So the covered surface is easily recognizable by the naked eye and can disturb obtaining plane and undamaged printings^24,27^. When morphology is relatively poor, it results in difficulties in obtaining a stable mixture and homogenous printings and surface covering^24^.

But, this work performs the anti-counterfeiting UCNPs working in real conditions. Thanks to perfectly stable colloids, we can use a commercial varnish and obtain a homogenous—invisible by naked eye transparent surface^28^. The nanoparticles spread in the varnish environment uniformly without any agglomeration. We have not used any surface modification with organic compounds or additional shells to obtain intense visible emission activated by two different excitation wavelengths and identified on a surface painted in any color^28–30^. Thanks to it, the obtained effect of the anti-counterfeiting materials is satisfying, but the structure is not so complicated as well as the synthesis process. Regarding future applications, it is a significant step to produce lighting marks quickly, fast, and most economically as possible^27,29^.

Until now, it has been possible to find several articles related to the inks emitting light applicable in anti-counterfeiting. There are research presenting inks excited with 980 nm, modified with quantum dots doped by Yb^3+^/Er^3+^or NaYF_4_:Ln^3+^@NaYF_4_, and NaYbF_4_:Er^3+^@NaYF_4_ activated by UV and NIR irradiation, but also with organic dyes excited in the UV region^31–35^. However, according to our knowledge, there is still a lack of studies about luminescent transparent varnish excited with NIR wavelength (980 nm and 808 nm) for security against falsification as an invisible in daylight cover for printings and different surfaces of valuable products.

Here, we present the luminescent anti-counterfeiting varnish modified with NaErF_4_:Tm^3+^@NaYF_4_ UCNPs to protect paper or other surfaces. This work shows the effect of co-doping the NaErF_4_ core with Tm^3+^ ions in the core@shell NaErF_4_:Tm^3+^@NaYF_4_ UCNPs and the improvement of UC intensity. The energy trapping centers formation, which reduce the energy migration to the internal lattice defect, is described^22,36^. The work performs not only the characteristic of the core@shell nanoparticles but also their promising application as a luminescent protection. We proved that anti-counterfeiting activity could be more efficient in the form of transparent varnish. It is an advantage compared to luminescent inks, which enable the modification of different surfaces independently on colorful printings. The article performs the UCNPs as an ideal additive for anti-counterfeiting varnish in terms of their specific spectroscopic features^6^. The obtained UCNPs enable the formation of unique color-based patterns and luminescent codes comparable to unique fingerprints, invisible by the naked eye, but possible to identify under a chosen excitation source in the UV to NIR range. Multiple excitation wavelengths, and specific shapes of emission spectra with sharp and well-defined intense bands, make the UCNPs competitive with luminescent carbon dots or organic dyes^33^. Moreover, previously reported upconverting protections, based on Yb^3+^ ions properties, are excitable by NIR radiation at around 975 nm only^7,37,38^. Still, the UCNPs described here provide an additional wavelength of excitation, 808 nm. Finally, the combination of varnish and UCNPs as its components allows for securing papers and other valuable goods, such as sculptures, paintings, clothing, footwear, electronics, and much more.

## Experimental section

### Materials

Oleic acid (extra pure, Fisher Scientific), 1-octadecene (technical grade 90%, Alfa Aesar), ammonium fluoride (min. 98.0%, Alfa Aesar), sodium hydroxide (95%, Sigma Aldrich), acetic acid glacial (99.5%, POCH S.A), acetic acid (80%, POCH S.A), yttrium oxide (99.9%, Intematix), erbium oxide, thulium oxide (99.9%, Stanford Materials), *n*-hexane (95%, VWR Chemicals), and ethanol (98.8%, POCH S.A.) were applied to synthesize nanoparticles. HR HIGH SLIP commercial UV varnish (FlintGroup, Konstantynow Lodzki, Poland) was used to prepare UCNPs modified finishing of prints.

### Apparatus

The measurements of the X-ray diffraction patterns (XRD) were conducted with the use of a Bruker AXS D8 Advance diffractometer (6–80°, 2θ range) with Cu K_α1_ radiation (*λ* = 1.5406 Å). The DLS (dynamic light scattering) was measured using a Malvern Zetasizer Nano ZS instrument. The HR-TEM (high-resolution transmission electron microscopy) and EDS (energy dispersive x-ray spectroscopy) images were collected using an FEI (S)TEM Titan G2 600–300 Hitachi HT7700 microscope to determine the morphology and average grain size distribution of the UCNPs.

The excitation sources were the fiber-coupled, solid-state diode-pumped (SSDP) continuous wave (CW) NIR 808 and NIR 975 lasers. Excitation spectra and luminescence decay curves were measured using an Opolette 355LD UVDM tunable laser (with a repetition rate of 20 Hz) and a 200 MHz Tektronix MDO3022 oscilloscope coupled to the Hamamatsu R928 photomultiplier detector and the QuantaMaster 40™ spectrofluorometer.

The photos of the varnishes and samples shown in Fig. 3 were taken with a Canon EOS 550D camera, with a sensitivity of ISO800, aperture f/4, and an exposition time of 1 s for the samples and 2.5 s for luminescent varnishes.

The varnish layer, with UPCNs, was applied by the IGT F1 apparatus. It simulated the printability via the flexographic or rotogravure technique, according to the ISO 12,647 standard.

### Core@shell synthesis

#### Synthesis of β-NaErF_4_:0.5%Tm^3+^ core NPs

Oleic acid, 1-octadecene (7.5 ml of oleic acid and 17.5 ml of 1-octadecene per 1 mmol of product), and 1 mmol of anhydrous rare-earth acetates were mixed and then placed in a three-neck round-bottom flask with a thermosensor and connected to the Schlenk-line. The solution was degassed on a vacuum at room temperature and heated to 120 °C for 60 min (< 1 mbar). Afterward, the clear solution cooled down to room temperature and was mixed with a methanol solution containing sodium hydroxide (2.5 mmol) and ammonium fluoride (4 mmol). The obtained suspension was stirred for 30 min at room temperature and then heated to complete the evaporation of methanol under N_2_ flow (around 80 °C). The following step was to heat the mixture to 100 °C and degas in a vacuum for 20 min. Finally, the solution was heated to 290 °C under N_2_ flow for 120 min and then cooled. The mixture was complete with ethanol (in a 1:1 ratio) and centrifuge (6000 rpm, 10 min) to separate the product. The precipitate was dissolved in hexane and centrifuged to separate by-products. In the end, an equal ethanol amount to the product solution was added to obtain the supernatant, and the precipitate was centrifuged. Ultimately, UCNPs were dried at room temperature or dissolved in hexane.

#### Synthesis of β-NaErF_4_:0.5%Tm^3+^@ β-NaYF_4_ core@shell NPs

Oleic acid and 1-octadecene with yttrium anhydrous acetate (core to shell ratio of 1:1 or 1:4; 7.5 ml of oleic acid and 17.5 ml of 1-octadecene per 1 mmol of product) were mixed in a three-neck round-bottom flask. Then the flask was connected to a Schlenk line. The solution was degassed by stirring under a vacuum (< 1 mbar) at 100 °C for 45 min. After degassing, the solution was heated to 150 °C for 45 min and cooled. Then, at room temperature, the core UCNPs were added to the solution (as a hexane colloid or dry powder). When the core was in hexane colloid form, the mixture was heated to 80 °C and degassed under vacuum for 20 min. The second method for powder core was heating to 100 °C and degassing under a vacuum for 20 min. Then, the solution was cooled to 50 °C. In the next step, the methanol solution of sodium hydroxide (2.5 or 1.3 mmol) and ammonium fluoride (4 or 5 mmol) was added and stirred for 20 min under N_2_ flow. Furthermore, the mixture was heated to 100 °C, degassed under N_2_ flow for 10 min, and degassed under vacuum for another 10 min. Finally, the solution was heated to 290 or 300 °C (see below for details) under N_2_ flow for 120/140 min and then cooled. The purification of the obtained NPs was the same as described for the core. Eventually, the UCNPs were dried, dissolved in hexane, and stored at 5–7 °C.

The core-to-shell ratio for NaErF_4_:Tm^3+^@NaYF_4__I synthesis was 1:1, and the Na:Y:F ratio in the shell precursor was 2.5:1:4. The core was added in hexane colloid, and the reaction was carried out at 290 °C for 120 min.

The core-to-shell ratio for NaErF_4_:Tm^3+^@NaYF_4__II synthesis was 1:4, and the Na:Y:F ratio in the shell precursor was 1.3:1:5. The core was added in powder form, and the reaction was carried out at 300 °C for 140 min.

### Preparation of varnish

Preparation of the printed paper samples covered with the layer of modified varnish involved two steps. In the first step, the samples of the modified varnish were prepared. The NaErF_4_:Tm^3+^@NaYF_4__I UCNPs were added to the varnish in the form of hexane colloid at a concentration of 120 mg/ml. The colloid was added to the varnish in amounts 5, 10, 30, and 40% of wt. to investigate the emission effect and printability properties. The second step was the application of the varnish using the laboratory flexographic machine IGT F1. This process was prepared according to ISO 12647. The appropriate amount of modified varnish was pipetted onto the anilox cylinder at the point of contact with the doctor's blade. Then four anilox revolutions were distributed over the entire surface and delivered to the cylinder containing the printing plate and from the printing plate to the printing substrate. The varnish layer was applied on the printed and unprinted paper with a grammage of 250 g/m^2^ to determine the emission of UCNPs printed in CMYK color paper. The IGT device was digitally controlled via a touch screen, enabling the mapping of the printing process. For the research presented, the anilox roller was used and characterized with a raster line of 90 l/cm and a paint transfer of 18 ml/m^2^. The printing plate contained a tint to obtain a solid layer of varnish on the paper, and the printing process was performed with a linear printing speed of 60 m/s. The pressure and angle of the doctor blade to the anilox cylinder were 120 N at 60 °C, and the pressure between the anilox roller and the printing plate was 100 N. Since the varnish used was UV finished, the printed test samples were fixed using a UV dryer Aktiprint IGT 12–1 device with the power of a UV lamp ABC-500 W.

## Results and discussion

### Structure and morphology of prepared NPs

**Figure 1:**
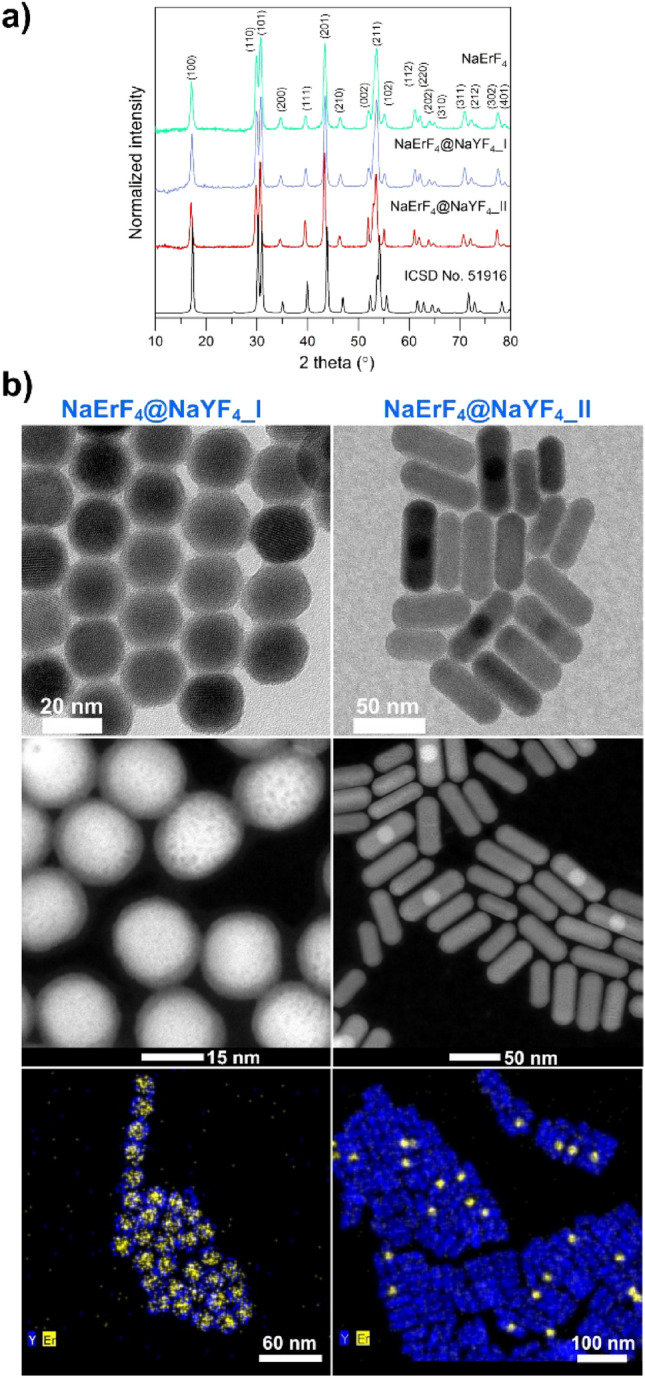
(**a**) XRD diffraction patterns of core and core@shell nanoparticles compared with the reference pattern of NaYF_4_ hexagonal phase form ICSD. (**b**) HR-TEM, HAADF, and EDS mapping images were taken for the two types of synthesized core@shell nanoparticles.

Two types of core@shell UCNPs based on fluoride matrices were synthesized using precipitation in the high-boiling solvents method. Slightly different procedure parameters described in  core@shell synthesis section allowed for producing UCNPs with different morphologies (i.e., spherical and rod-like shapes [see Fig. 1]). Both, NaErF_4_:Tm^3+^@NaYF_4__I and NaErF_4_:Tm^3+^@NaYF_4__II structures crystallized as hexagonal phases, and their X-ray diffraction (XRD) patterns are presented in Fig. 1a. The diffraction peaks of the core and the core@shell structures align well with the reference pattern of β-NaYF_4_ (ICSD No. 51916). No additional lines were observed, which confirms the samples have no impurities. The slight shift of the XRD lines into the lower values of 2*θ* degrees, compared with the reference pattern, resulted from different radii of RE ions in a matrix structure, where *r*(Y^3+^) = 1.019 and *r*(Er^3+^) = 1.004 (for the coordination number CN = 8)^39^.

**Figure 2:**
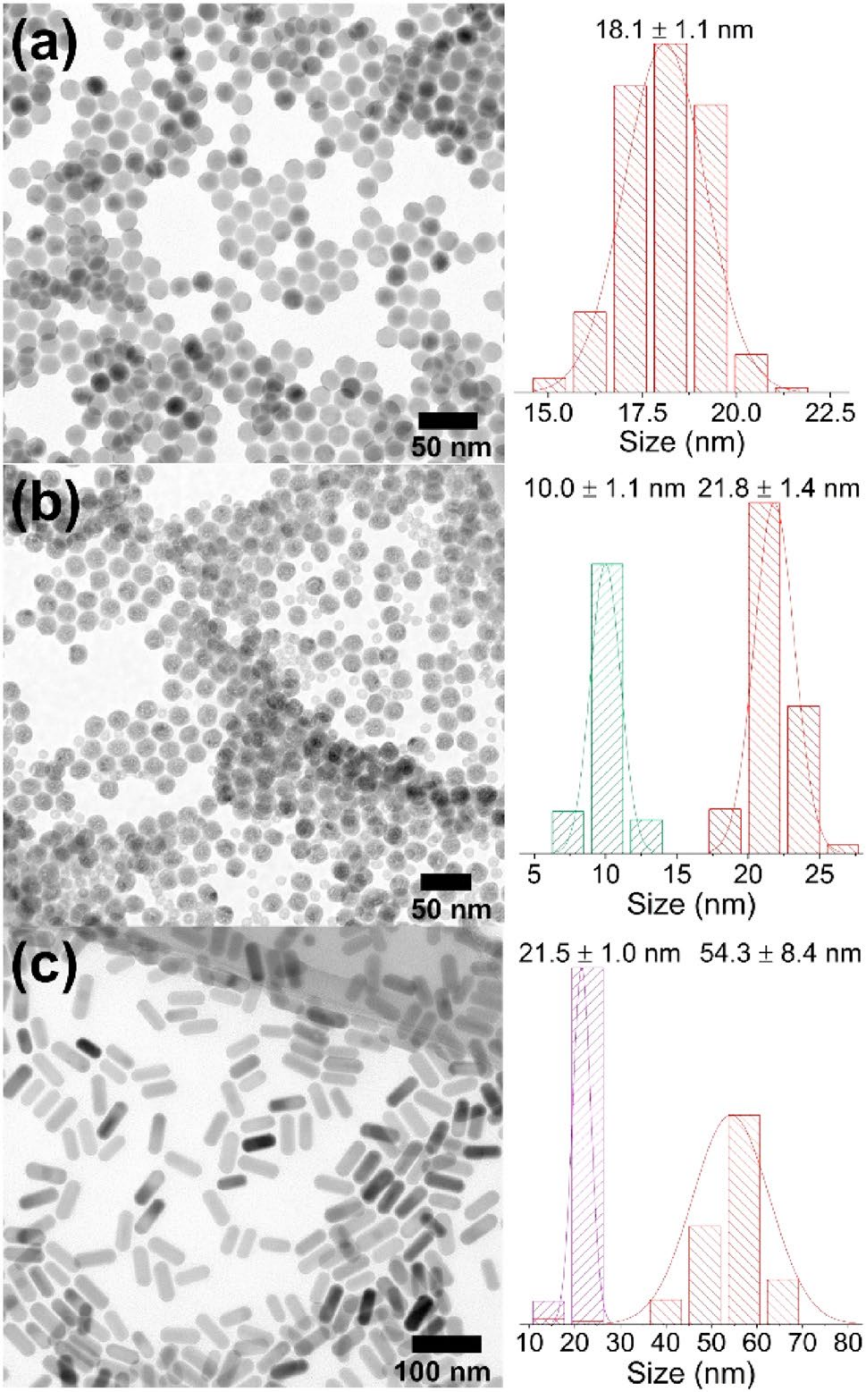
TEM images with NPs size distribution for (**a**) NaErF_4_:Tm^3+^ core UCNPs, (**b**) NaErF_4_:Tm^3+^@NaYF_4__I NPs, and (**c**) NaErF_4_:Tm^3+^@NaYF_4__II UCNPs.

The crucial criterion for determining the quality of a core@shell structure is its average grain size and size distribution. Here, the TEM images helped to establish these parameters (Fig. 2). The average grain size of the core UCNPs was 18.0 ± 1.1 nm. This narrow size distribution (Fig. 2a) was advantageous in the initial step for further core@shell preparation. The TEM images of the first core@shell structure NaErF_4_:Tm^3+^@NaYF_4__I (Figs. 1b and 2b) illustrate two different fractions of the UCNPs. The larger ones have an average grain size of 21.8 ± 1.4 nm, while the smaller of 10.0 ± 1.1 nm. This result indicates the presence of some unreacted residues, though in low quantity, which was not possible to detect with XRD analysis. However, TEM images confirmed that the core@shell synthesis was still efficiently performed. The TEM images of the second structure NaErF_4_:Tm^3+^@NaYF_4__II display only one fraction of UCNPs, with a relatively narrow size distribution (Figs. 1b and 2c, length 54.3 ± 8.4 nm, width 21.5 ± 2.0 nm). The increasing average grain size of the UCNPs after shell covering is visible for the I and II structure, which is consistent with DLS histograms and core@shell structure formation (Fig. S1). Moreover, the shape of crystallites differs, and the UCNPs type I and type II are spherical and rod-like, respectively. Pan et al.^40^ and Lin et al.^1^ likewise observed the rod-like shape of UCNPs based on the NaErF_4_ matrix. However, their observations (i.e., the rod-like form is favorable for the core@shell structure, in which the ratio between RE^3+^ ions and F^-^ ions in the shell is 1:4; for a higher ratio, 1:5.6, spherical particles are present) are opposite ours. Here, the results indicate that the ratio of 4 for RE^3+^ ions to F^-^ resulted in a spherical shape (structure I), but a proportion of [RE]/[F] greater than or equal to 5 allowed for obtaining rod-like UCNPs. The above analysis confirms that the ratio between the core and shell precursors, in addition to the reaction temperature and time of the synthesis, greatly influences the final morphology of the UCNPs.

More detailed characterization of the UCNPs structure HR-TEM, STEM (HAADF, high-angle annular dark-field), and EDS mapping were employed (Fig. 1b). The HAADF technique proves a bright core area and darker shell layer region of NaErF_4_:Tm^3+^@NaYF_4__I UCNPs. EDS mapping shows the presence of Er^3+^ ions in the core and Y^3+^ ions in a shell layer. However, for smaller UCNPs, only Y^3+^ ions are visible, which confirms that the minor part of UCNPs is unreacted shell particles.

Interestingly, for rod-like UCNPs, the bright, spherical cores containing Er^3+^ ions are visible in only the center of some particles and are covered by a darker shell with Y^+^ ions. The remaining rod-like UCNPs contain Y^3+^ ions and a small amount of Er^3+^ ions. It means that the formation of the core@shell structures in the form of rods is not homogenous, as in the case of spherical particles. The other ions, such as Na^+^ and F^-^, are evenly distributed in all particles and the I and II structures.

### Spectroscopic properties of core@shell UCNPs

**Figure 3:**
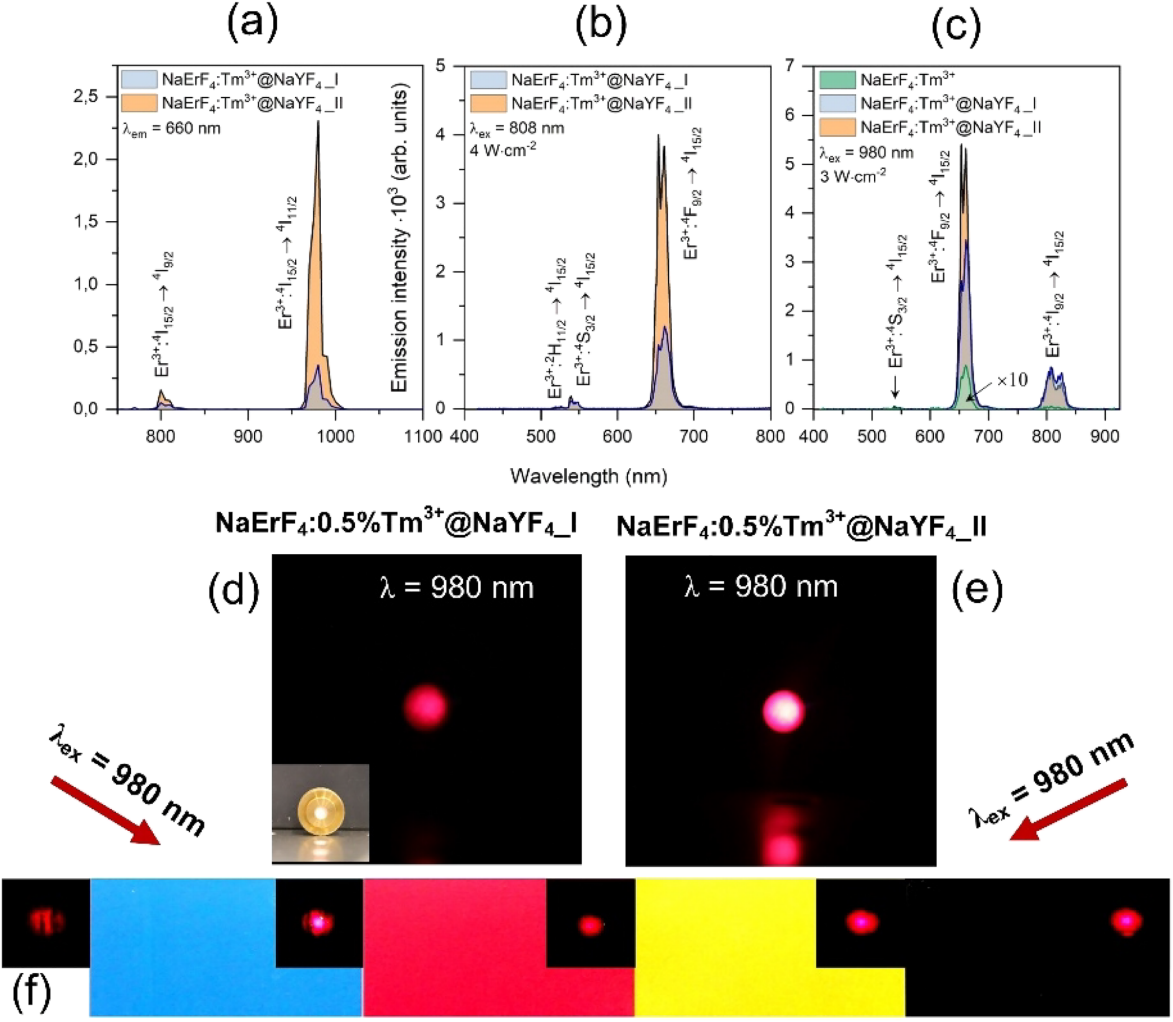
(**a**) Excitation spectra in the NIR range of the synthesized nanoparticles and their emission under (**b**) 808 nm and (**c**) 980 nm excitations; (**d**,**e**) emission of nanoparticles in the form of powders, (**f**) emission from UCNPs embed into transparent varnishes used to cover selected ink colors.

Up-converting properties of the NaErF_4_:Tm^3+^ core and NaErF_4_:Tm^3+^@NaYF_4_ core@shell UCNPs were characterized based on luminescence measurements under 808 and 980 nm laser excitation wavelengths. The excitation spectra within the NIR range (see Fig. 3a) consist of the transition bands characteristic for Er^3+^ ions, assigned to ^4^I_15/2_ → ^4^I_9/2_ at around 802 nm, ^4^I_15/2_ → ^4^I_11/2_ at 979 nm, which indicates the possibility of the bimodal method of excitation under NIR radiation. The band's intensity, with a maximum of around 979 nm, is the strongest for both structures.

The presented emission spectra shows transition bands characteristic of Er^3+^ ions (^2^H_11/2_ /^4^S_3/2_ → ^4^I_15/2_, ^4^F_9/2_ → ^4^I_15/2_, ^4^I_9/2_ → ^4^I_15/2_) (Fig. 3b,c). However, green bands are almost unnoticeable under a 980 nm excitation wavelength. It is worth noting that the great enhancement in the luminescence intensity for I and II core@shell structures concerning the core, most prominent for ^4^F_9/2_ → ^4^I_15/2_ emission band, is noticed (Fig. S2). It is a consequence of the luminescent core protection by the inert shell and simultaneously limiting the energy migration to surface defects.

The NaErF_4_:Tm^3+^@NaYF_4_ core@shells exhibited strong red luminescence, corresponding to the ^4^F_9/2_ → ^4^I_15/2_ transition under 808 and 980 nm even under low laser powers (2.5–4 W cm^−2^), visible in Fig. 3d–f. Meanwhile, the domination of emission from the ^4^F_9/2_ state is observed with integrated emission intensities of the observed bands (Fig. S3). Furthermore, in Fig. S4, the chromaticity diagrams are presented, and the x and y coordinates show the changes in emission color about the excitation wavelength. For 980 nm excitation, the coordinates for both compounds are similar, and their position is in the red region. However, 808 nm excitation shifted the emission to the orange area, particularly for NaErF_4_:Tm^3+^@NaYF_4_ _I sample. This dependence is an effect of the UC mechanism discussed below.

The measurements of luminescence lifetimes complete the spectroscopic properties analysis and upconversion mechanism determination (Fig. S5, Table S1). Based on the calculated lifetimes, the first conclusion is the ^2^H_11/2_ → ^4^I_15/2_ transition is responsible for the longest lifetime in the case of both structures: I and II. On the contrary, the lowest calculated values of the lifetimes measured under 980 nm laser excitation were for the ^4^F_9/2_ → ^4^I_15/2_. The reason is that Tm^3+^ ions play the role of trapping centers and mediate the UC mechanism^41^. Generally, all the luminescence lifetimes were longer for the NaErF_4_:Tm^3+^@NaYF_4__II structure.

**Figure 4:**
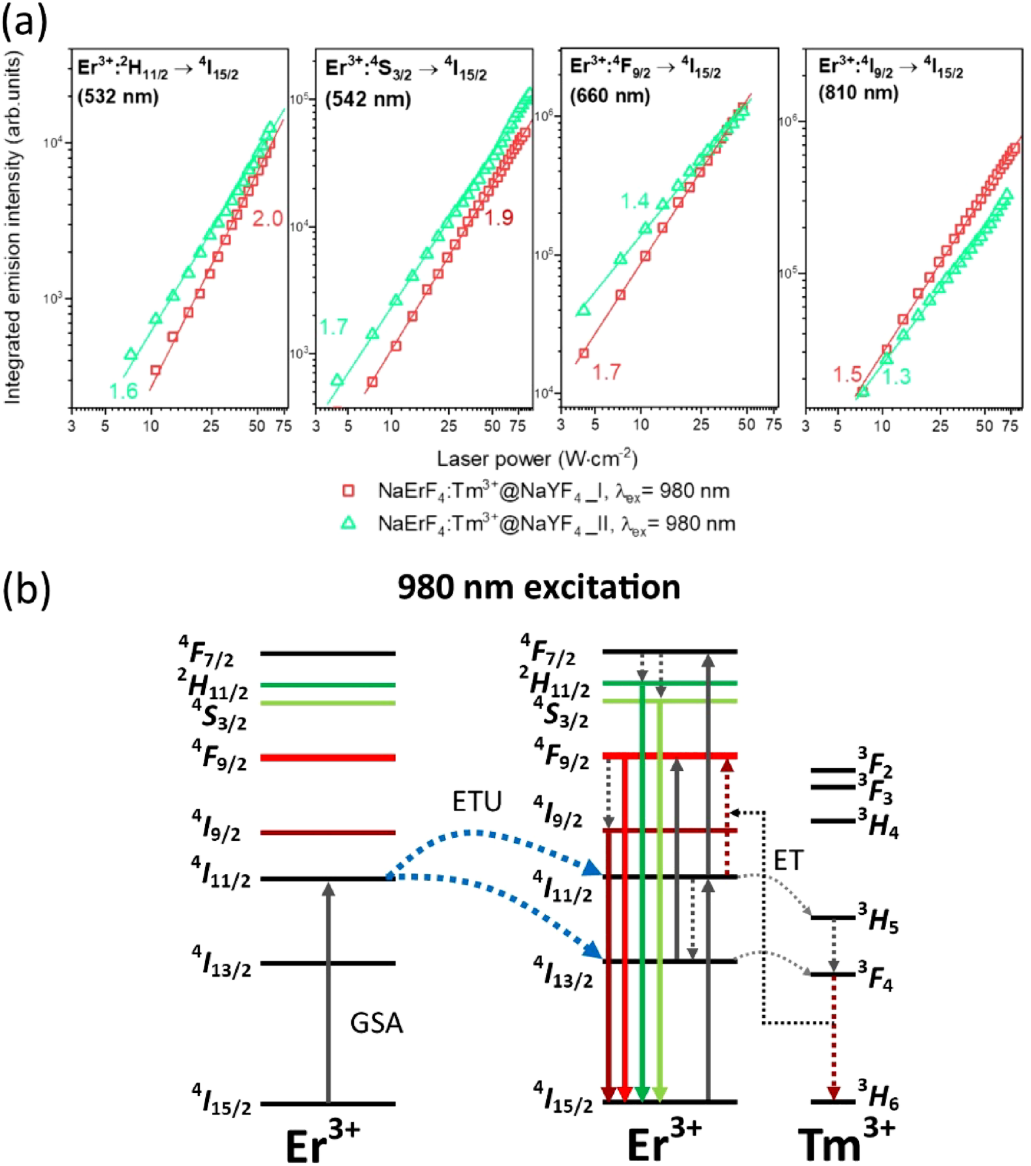
(**a**) Double-logarithmic plots of the upconversion emission intensity versus laser power density, measured for the NaErF_4_:Tm^3+^@NaYF_4_ NPs and (**b**) proposed UC mechanisms responsible for the observations under 980 nm laser excitation.

Analysis of the emission and excitation spectra, together with luminescence lifetimes and slopes of the up-conversion emission intensity versus laser power density, is the basis for the UC mechanism determination (Fig. 4a,b). When we consider the theoretical mechanism of Er^3+^ ions UC under 980 nm excitation, several processes are possible. It is ground state absorption (GSA) to the ^4^I_11/2_ energy level via single-photon absorption followed by excited-state absorption (ESA) with the second photon absorption to the ^4^F_7/2_ level. However, in highly Er^3+^-doped samples population to the higher excited states occurs mainly by energy transfer (ETU) between Er^3+^ ions^34^. After a sequence of GSA and ETU processes and non-radiative relaxations, Er^3+^ ions are excited to the ^2^H_11/2_ or ^4^S_3/2_ state yielding emission bands in the green part of the spectrum. However, after absorption of the first photon, non-radiative relaxation to the ^4^I_13/2_ level within the same ion or CR to neighborhood Er^3+^ ions can also appear^36^. In sequence, absorption of a second photon via ETU or ESA to the ^4^F_9/2_ energy level and relaxation with red emission can occur. The intense red emission of the prepared samples, suggest that Er^3+^ ions are populated mainly to their ^4^F_9/2_ state or other energy levels are effectively quenched. The mechanism of Er^3+^ ions population is strongly affected by the Tm^3+^ ions present in the structure of nanoparticles. Tm^3+^ ions play the role of "energy trapping" centers^42,43^. ET and CR between dopants depopulate excited states of Er^3+^ ions, resulting in Tm^3+^ ions in their ^3^F_4_ excited state. Next, the ET from Tm^3+^ ions to the Er^3+^ ions is possible resulting in Er^3+^ ions in their ^4^F_9/2_ excited state in greater amount than if there were no Tm^3+^ ions in the system^35–38^.

### Properties of modified varnish

The research related to the modification of the varnish with a colloid containing the UCNPs presented in this article revealed that even at the lowest, namely 5% wt of colloid added to the varnish layer applied to the paper, the emission of light is clearly visible. The bright red luminescence occurs from a piece of paper covered with CMYK prints covered by luminescent varnish (Fig. 3f).

The significant result is that the addition of colloids did not alter the spectral data, the perception of the color of the print under the varnish layer, or the visual impression of the varnish itself (Tables S2, S3, and Fig. S7). Moreover, no negative influence of the addition of colloid to the varnish was observed, even in 40% wt, when covering the print samples with varnish. Furthermore, introducing UCNPs as a colloid ensures good nanoparticle dispersion in the varnish layer, even in high amounts. The proposed method has a significant advantage because the varnish does not contain any coloring substances. There is no factor that could cause quenching of the emission of UCNPs, which we have observed when incorporating them into some printing inks. Additionally, the varnish with UCNPs can be applied to the print surface selectively in a given place of the pattern on the print when finishing the packages. Details of the performed tests and the in-depth results are available in the SI (1.3 Methodology of testing the properties of samples covered with luminescent varnish, Tables S2, S3, Fig. S7).

## Conclusions

The precipitation reaction in the high-boiling point solvents made it possible to synthesize the UCNPS based on fluoride matrix NaErF_4_:Tm^3+^@NaYF_4_ and with core@shell structure. The suitable selected conditions of synthesis resulted in spherical and rod-like nanoparticles. Differences in the shape of UCNPs allowed for their spectroscopic properties comparison. The careful structural and luminescence analysis showed that rod-like NaErF_4_:Tm^3+^@NaYF_4__II nanoparticles have better spectroscopic properties. They exhibited much more intense emission with higher chromaticity of the red emission color. At the same time, spherical NaErF_4_:Tm^3+^@NaYF_4__I were more suitable for dispersion in the varnish used for color print covering. Generally, the obtained products presented pure red upconversion under 808 and 980 nm laser excitations. Using Tm^3+^ ions as a co-dopant influenced the excitation mechanism by the supporting ^4^F_9/2_ excitation state of Er^3+^ ions. The most crucial result of this work is the preparation of the transparent varnish, modified with NaErF_4_:Tm^3+^@NaYF_4_ core@shell UCNPs. According to our knowledge, it was the first time that luminescent prints covered by bimodal up-converting varnish were examined.

## Supplementary Information

Supplementary Information.

## Data Availability

The datasets used and/or analysed during the current study available from the corresponding author on reasonable request.
