# Supplementary material for: Anti-counterfeiting system based on luminescent varnish enriched by NIR- excited nanoparticles for paper security
Source: Sci Rep. 2022 Nov 12;12:19388. doi: 10.1038/s41598-022-23686-9 (PMC9653438; doi:10.1038/s41598-022-23686-9)
Supplement: Supplementary file 1 — Supplementary Information. [file 41598_2022_23686_MOESM1_ESM.pdf]

Supporting information

for

Anti-counterfeiting system based on luminescent varnish enriched by NIR-excited nanoparticles for paper security

D. Przybylska,[a] T. Grzyb[*a], A. Erdman,[b] K. Olejnik,[b] A. Szczeszak,[*a]

[a]Adam Mickiewicz University in Poznań, Faculty of Chemistry, Department of Rare Earths, Uniwersytetu Poznańskiego 8, 61614 Poznan, Poland

[b]Łódź University of Technology, Centre of Papermaking and Printing, Wólczańska 221, 93-005 Łódź, Poland

1. Experimental

1.1. Synthesis of anhydrous rare-earth acetates

With rare earth oxides, rare earth anhydrous acetates were prepared. 30 mmol of erbium oxide was mixed with acetic acid (120 ml) and distilled water (120 ml) in a 500 ml three-neck round-bottom flask. Then the mixture was heated at 95 °C under a reflux condenser to dissolve the oxide and obtain a transparent solution (around 15 h). After cooling to room temperature, the solvent was evaporated with a rotavapor, almost to a dry powder. Afterward, 80 ml of acetic acid were added and stirred, heating at the same time at around 60 °C on the rotavapor. Dissolved erbium hydrous acetate was transferred to a three-neck round-bottom flask with a dropping funnel and heated to 120 °C under a reflux condenser. After reaching the appropriate temperature, 20 ml of anhydrous acetic acid were added via a dropping funnel to the solution. The temperature above the boiling point of glacial acetic acid (around 120 °C) was maintained for 2 h. Then, after the mixture was cooled to room temperature, the solvent was removed with a rotavapor to obtain a dry powder. TGA analysis was performed to check the residual water content for the final product. Ytterbium and erbium anhydrous acetates were prepared analogously.

1.2. Methodology of lifetime calculation

To estimate the average (effective) lifetimes of the observed excited state of Er^{3+}, the presented equation, determined by the non-exponential character of luminescence decays, was used for lifetimes calculation:

$$\tau = \frac{\int_0^\infty tI(t)dt}{\int_0^\infty I(t)dt}$$

where τ is the decay time, and *I(t)* is the intensity at time *t*.[1]

The lifetime was determined based on the integration area under normalized decay curves, with the mathematical type of the area. OriginLab 2022 was used for the calculations.

1.3 Methodology of testing the properties of samples covered with luminescent varnish

Gloss was measured using a ZGM 1020 gloss meter, which tests the full range of gloss from matt surfaces to very high gloss elements (compliant with ISO 2813, ASTM D 523). This device enables measurements using a head with a geometry of 75 O with an accuracy of less than 1 GU gloss unit. The device was calibrated using a calibration standard. Then the gloss of the layers of varnish applied on the unprinted substrate and on each of the individual colors of the print was examined.

Optical density and spectral measurements in the CIE L * a * b * space were performed using the X-Rite Exact spectrophotometer, which, due to the possibility of carrying out both measures in one instrument, is also called a spectrodensitometer.

The color difference (ΔE) was estimated for printed samples covered with varnish with different amounts of luminescent colloid (5%, 10%, 30%, and 50%), in which measurements concerned a particular color: cyan, magenta, yellow, and black. The size of the color deviation of a given sample in relation to the reference sample (which is not included) is determined as the dependence of the addition of luminescent nanoparticles to the varnish regarding the color change. The reference sample was paper printed with CMYK colors with a varnish layer without the addition of UCNPs - this point of reference will allow for observing whether this addition might affect the color change of the final product refined with varnish. The average values of the parameters L * (brightness), a * (red-green axis), and b * (blue-yellow axis) from which the total difference value ΔE_{ab} was calculated.

2. Structure and morphology of synthesized nanoparticles

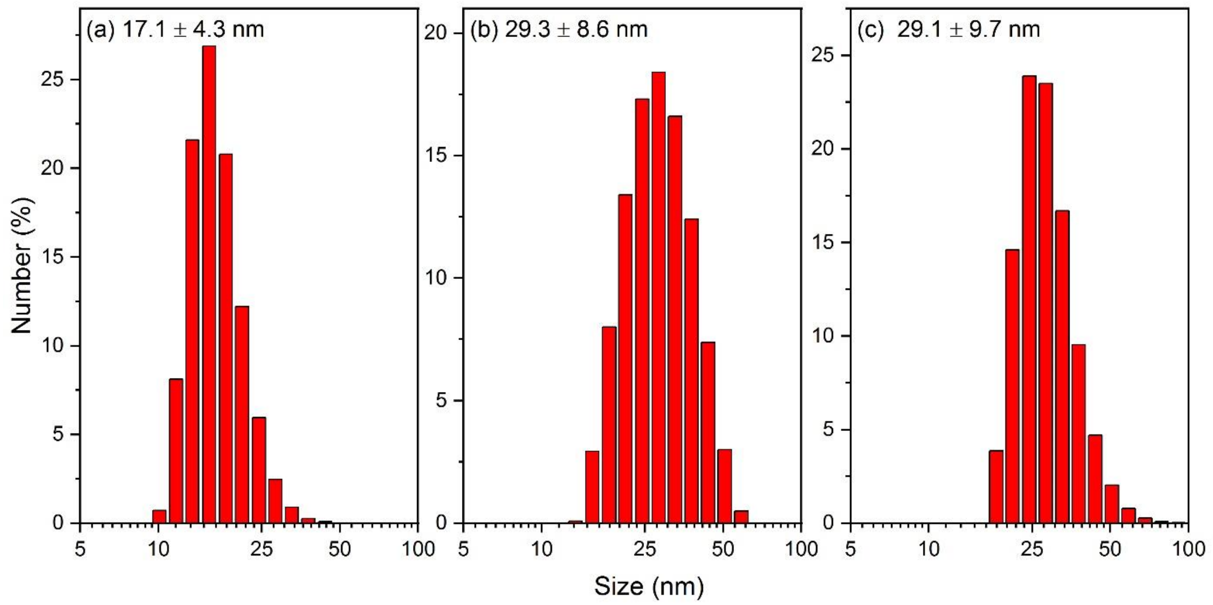

Fig. S1. Histogram of size distribution of core and core@shell NPs, in which (a) $NaErF_4:Tm^{3+}$, (b) $NaErF_4:Tm^{3+}@NaYF_4$_I, and (c) $NaErF_4:Tm^{3+}@NaYF_4$_II were obtained from DLS measurements.

3. Spectroscopic properties of prepared nanoparticles

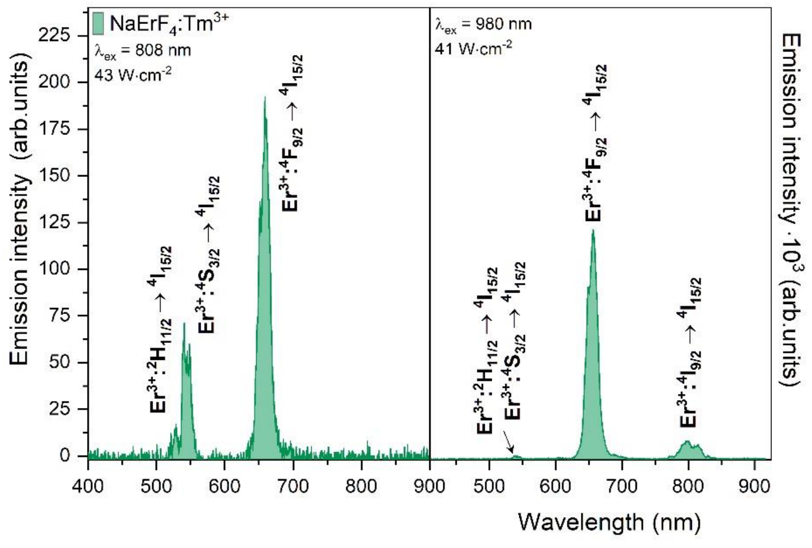

Fig. S2. Emission spectra of $NaErF_4:Tm^{3+}$ core UCNPs under (a) 808 nm and (b) 980 nm excitations.

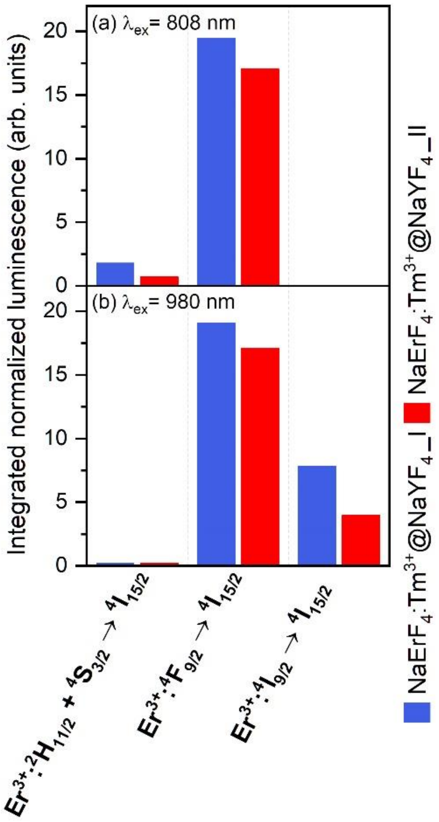

Fig. S3. Integrated emission intensities of $NaErF_4:Tm^{3+}$@$NaYF_4$_I (blue) and $NaErF_4:Tm^{3+}$@$NaYF_4$_II (red) NPs after normalization under (a) 808 nm and (b) 980 nm excitation wavelengths.

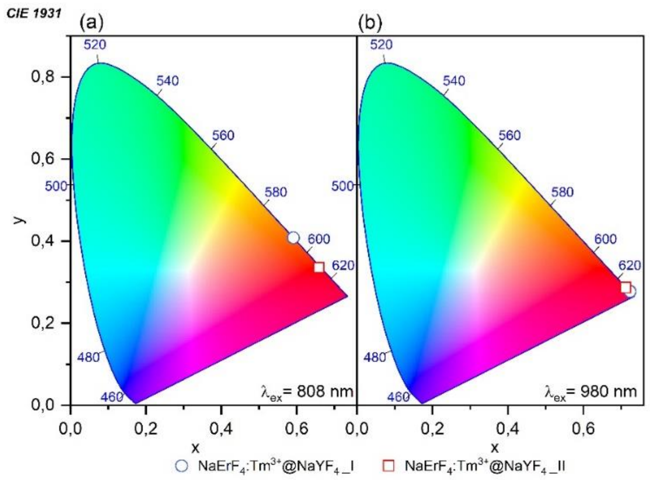

Fig. S4. CIE chromaticity diagrams of $NaErF_4:Tm^{3+}$@$NaYF_4$_I (blue circle) and $NaErF_4:Tm^{3+}$@$NaYF_4$_II (red square) under (a) 808 nm, (b) 980, and (c) 1532 nm excitation wavelengths.

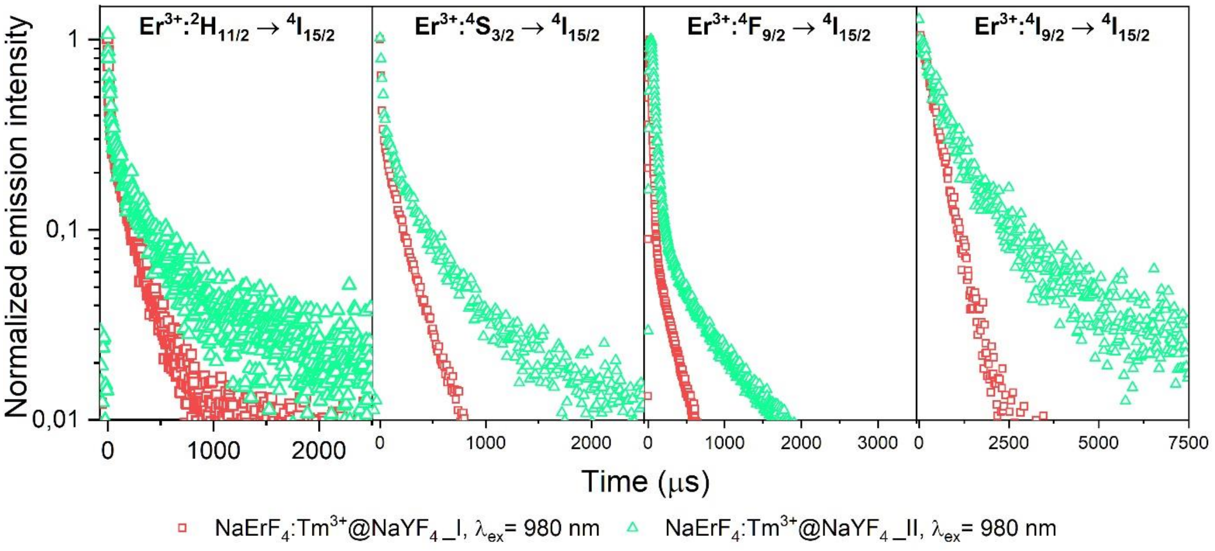

Fig. S5. Luminescence decays recorded for $NaErF_4:Tm^{3+}$@$NaYF_4$ UCNPs under 980 nm.

Table S1. Emission lifetimes calculated from the measured luminescence decays for $NaErF_4:Tm^{3+}$@$NaYF_4$_I and $NaErF_4:Tm^{3+}$@$NaYF_4$_II UCNPs under 980 nm laser excitation (for decays, see Fig. S6).

	Excitation wavelength	Er^{3+}:$^2H_{11/2} \rightarrow {}^4I_{15/2}$	Er^{3+}:$^4S_{3/2} \rightarrow {}^4I_{15/2}$	Er^{3+}:$^4F_{9/2} \rightarrow {}^4I_{15/2}$	Er^{3+}:$^4I_{9/2} \rightarrow {}^4I_{15/2}$
		Average lifetime (μs)			
**$NaErF_4$: Tm^{3+}@ $NaYF_4$_ I**	980 nm	85.7	64.8	49.8	477.5
**$NaErF_4$: Tm^{3+}@ $NaYF_4$_ II**	980 nm	156.5	154.9	115.7	1051.6

4. Properties of paper samples covered with varnish contain luminescent colloids of $NaErF_4:Tm^{3+}$@$NaYF_4$_I UCNPs

For the paper samples covered with varnish, the following tests were performed:

Gloss measurement

Table S2 Gloss value for printed paper in CMYK color, covered by varnish with the addition of different amounts of luminescent colloid with $NaErF_4:Tm^{3+}$@$NaYF_4$_I UCNPs (varnish type: TTR GLOSS, SOFT TOUCH, HR HIGH SLIP).

		No print	C	M	Y	K
**No varnish**		**12.7**	**55.9**	**61.4**	**57.8**	**70.9**
**TTR GLOSS**	0%	13.4	18.4	20.7	18.2	31.2
	5%	13.8	20.3	21.6	20.4	39.0
	10%	13.1	17.8	22.9	17.6	32.6
	30%	13.2	19.3	24.6	19.1	33.5
	40%	12.4	18.1	26.0	21.5	40.2
**SOFT TOUCH**	0%	14.0	18.5	21.0	22.8	27.8
	5%	14.5	22.6	28.0	25.9	31.5
	10%	14.4	24.1	27.0	24.5	30.4
	30%	16.5	24.4	30.3	25.2	34.1
	40%	16.2	23.9	26.6	25.8	35.7
**HR HIGH SLIP**	0%	13.0	15.0	22.3	20.2	32.1
	5%	13.2	15.5	21.4	15.8	33.3
	10%	14.2	16.8	21.6	19.0	32.5
	30%	13.5	16.3	22.1	20.2	34.0
	40%	15.2	21.4	32.8	23.2	45.8

Based on the obtained results presented in the above table, it can be observed that the varnishes covering printed paper samples affect the optical perception of the print, thereby significantly decreasing the gloss value. The gloss of the prints without a varnish layer has a different value, which depends on the tested color, potentially affecting the gloss value of the varnish layer applied to the print. However, it was noted that the content and concentration of luminescent nanoparticles do not affect the gloss value of the prints but rather depend on the printed color and type of varnish.

Optical density

Table S3. The optical density values for printed paper in CMYK color, covered by varnish with the addition of different amounts of luminescent colloid with $NaErF_4$:Tm@$NaYF_4$_I UCNPs (varnish type: TTR GLOSS, SOFT TOUCH, HR HIGH SLIP).

		C	M	Y	K
**NO VARNISH**		**1.37**	**1.45**	**1.33**	**1.83**
**TTR GLOSS**	0%	1.23	1.28	1.21	1.61
	5%	1.23	1.30	1.22	1.68
	10%	1.23	1.31	1.22	1.68
	30%	1.25	1.32	1.24	1.69
	40%	1.24	1.33	1.25	1.73
**SOFT TOUCH**	0%	1.15	1.18	1.11	1.57
	5%	1.24	1.32	1.25	1.70
	10%	1.26	1.32	1.27	1.70
	30%	1.26	1.35	1.27	1.76
	40%	1.27	1.35	1.28	1.78
**HR HIGH SLIP**	0%	1.21	1.29	1.23	1.57
	5%	1.22	1.29	1.22	1.63
	10%	1.23	1.29	1.22	1.65
	30%	1.24	1.31	1.24	1.65
	40%	1.29	1.37	1.26	1.81

The slight changes in the optical density value for given colors after covering a varnish layer with and without luminescent colloid are noticeable. The obtained values in the case of printing without varnish are higher than after coating with varnishes (Table S3), which might be due to the thickness of the applied layer of varnish, its properties, and the properties of the paper used for testing. As observable in the diagram above (Fig. S7), a linear increase in optical density can be seen, along with an increase in the luminescent colloid content in the varnish; however, the presented value differences are negligible.

Color difference (ΔE)

Depending on the obtained color deviation, five ranges of the degree of identification of the color difference can be distinguished:

• ΔE < 1: color difference imperceptible to the human eye,

• ΔE = 1 ÷ 2: color difference visible to an experienced person,

• ΔE = 2 ÷ 3.5: color difference visible to the observer regardless of experience,

• ΔE = 3.5 ÷ 5: very clear color difference,

• ΔE > 5: color difference proving the distinctiveness of the measured colors.

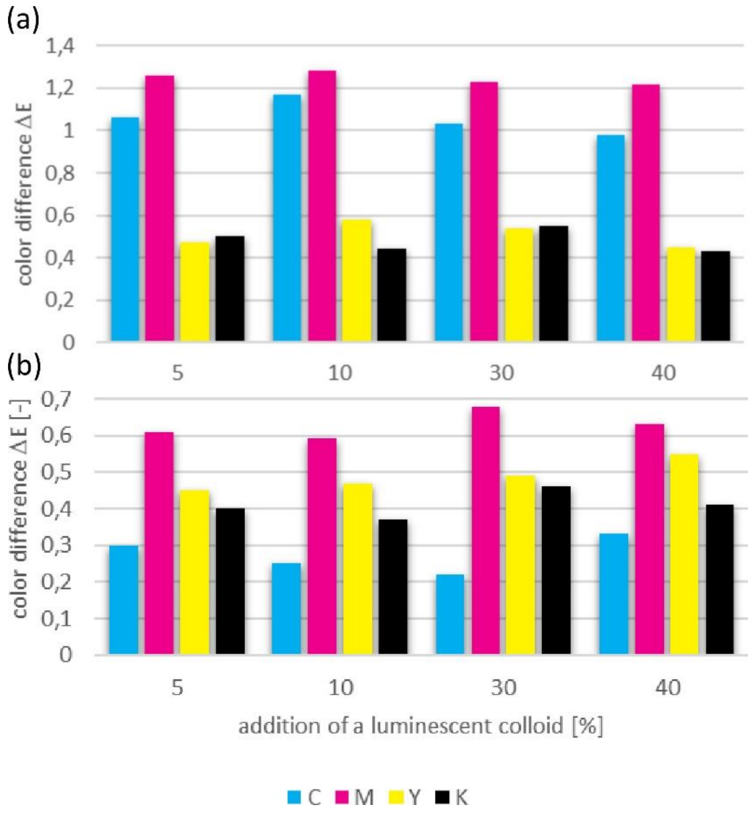

Fig. S7 Color difference for printed paper in CMYK color, covered by varnish with the addition of different amounts of luminescent colloid with $NaErF_4$:Tm@$NaYF_4$_I UCNPs, with (a) SOFT TOUCH and (b) TTR GLOSS varnish.

Adding luminescent nanoparticles to the varnish causes a slight change in the reception of individual colors, depending on the type of varnish used. For example, for the Soft Touch varnish covered on the cyan and magenta colors, ΔE is greater than 1. In contrast, the slightest color deviation (ΔE of 0.5) is noticeable for yellow and black colors. The most significant color

difference for the TTR Gloss varnish is noticeable for magenta (ΔE of about 0.6) and yellow. As observable in the above graph, only in the case of the Yellow color is there a linear increase in the color deviation about the reference sample with the rise of the luminescent colloid concentration. Notably, the different concentrations of nanoparticles do not affect the perception of the visible color—color deviations for various phosphor additives in the varnish are maintained at a similar level.
